# Multimodal Imaging of Oncocytic Lipoadenoma Arising from the Parotid Deep Lobe with Medial Extension into the Parapharyngeal Space: A Case Report with Histopathologic Findings and Literature Review

**DOI:** 10.3390/diagnostics16091366

**Published:** 2026-04-30

**Authors:** Jong-Uk Lee, Hye Jin Baek, Kwang Ho Choi, Eun Cho, Hyo Jung An

**Affiliations:** 1 Department of Radiology, Research Institute for Convergence of Biomedical Science and Technology, Pusan National University Yangsan Hospital, Pusan National University School of Medicine, 20 Geumo-ro, Mulgeum-eup, Yangsan-si 50612, Republic of Korea; 2 Department of Thoracic and Cardiovascular Surgery, Research Institute for Convergence of Biomedical Science and Technology, Pusan National University Yangsan Hospital, Pusan National University School of Medicine, 20 Geumo-ro, Mulgeum-eup, Yangsan-si 50612, Republic of Korea; 3 Department of Radiology, Gyeongsang National University School of Medicine, Gyeongsang National University Changwon Hospital, 11 Samjeongja-ro, Seongsan-gu, Changwon 51472, Republic of Korea; 4 Department of Pathology, Busan Paik Hospital, Inje University College of Medicine, Bokji-ro 75, Busanjin-gu, Busan 47392, Republic of Korea

**Keywords:** oncocytic lipoadenoma, parotid gland, computed tomography, magnetic resonance imaging, ultrasonography

## Abstract

**Background**: Oncocytic lipoadenoma is an exceptionally rare benign fat-containing salivary gland tumor that most commonly arises in the parotid gland. Previous case reports have largely focused on histopathology with limited or single-modality imaging documentation; therefore, practical preoperative radiological characterization remains challenging. **Case Presentation**: A 46-year-old male presented with a 2-year history of a slowly enlarging right-sided parotid mass. Computed tomography and magnetic resonance imaging showed a well-circumscribed fat-containing mass with a discrete medially enhancing solid component, mild diffusion restriction and small cystic foci without aggressive features. Ultrasonography revealed a heterogeneously hypoechoic parotid mass; however, limited acoustic penetration hindered evaluation of the deep portion. A core-needle biopsy was inconclusive, and an atypical lipomatous tumor could not be excluded. Subsequent surgical excision confirmed an oncocytic lipoadenoma, a biphasic tumor comprising mature adipose tissue and cytokeratin 7-positive oncocytic epithelial nests. The patient has remained recurrence-free for 7 years after surgery. **Conclusions**: Fat-containing parotid tumors can be diagnostically challenging because imaging findings are often nonspecific, and biphasic lipoepithelial entities are rarely encountered. This case highlights that awareness of the pattern of macroscopic fat with a discrete enhancing non-fat component, interpreted alongside histopathological findings, may help narrow the differential diagnosis, guide management, and reduce diagnostic uncertainty.

## 1. Introduction

The World Health Organization Classification of Head and Neck Tumors provides an overarching framework for salivary gland tumor taxonomy, within which these rare fat-containing variants are recognized [1]. Neoplastic fat-containing (lipomatous) tumors of the salivary glands are uncommon, accounting for approximately ≤0.5% of all salivary gland neoplasms [1]. Based on their histological composition, these lesions can be broadly divided into monophasic adipocytic neoplasms, such as lipoma and its variants, and biphasic (hybrid lipoepithelial) lesions, in which salivary epithelial components are admixed with a variable adipose component [2]. Within the biphasic category, entities are further classified according to the nature of the epithelial component, including sialolipoma and oncocytic lipoadenoma. Notably, oncocytic lipoadenoma has been reported under varying nomenclature (e.g., oncocytic sialolipoma) in the literature [2].

Oncocytic lipoadenoma is an exceptionally rare, benign salivary gland tumors characterized by an admixture of oncocytic epithelial elements and mature adipose tissue that most commonly arise in the parotid gland [3,4]. To date, only a limited number of cases have been published, most of which have primarily emphasized histopathological, cytological, immunohistochemical, or genetic findings to establish diagnostic criteria [3,4,5,6,7,8,9,10,11,12,13,14,15,16,17,18]. Although imaging findings have been described, radiologic reports have often been limited to a single modality or brief descriptions, and the reported imaging features remain heterogeneous across publications [3,4,5,6,7,8,9,10,11,12,13,14,15,16,17,18]. Furthermore, when figures are provided, they are often limited in number or technical detail, restricting practical radiologic–pathologic correlation and making preoperative radiologic characterization challenging in routine practice [9,11,14,16,17,18].

To address this gap in the literature, we report a case of parotid oncocytic lipoadenoma and describe comprehensive, high-quality multimodal imaging findings, including ultrasonography (US), computed tomography (CT), and magnetic resonance imaging (MRI), along with the histopathological features. In this case report, we emphasize the clinical value of a multimodal imaging approach for the preoperative evaluation of fat-containing parotid masses.

## 2. Case Report

**Figure 1:**
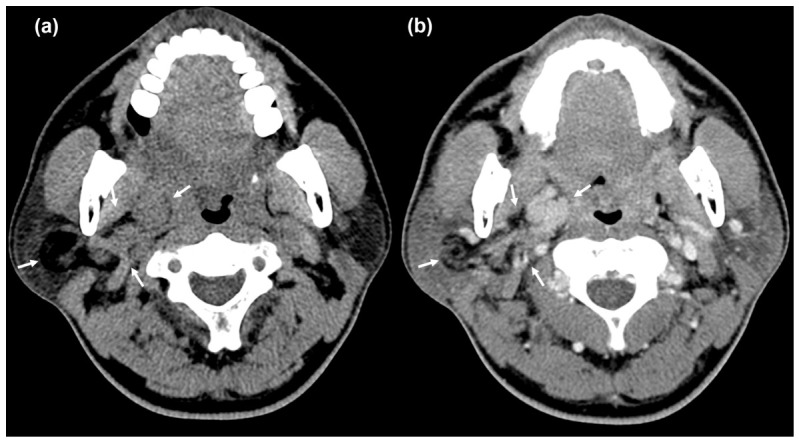
**CT findings.** (**a**) Axial non-contrast CT shows a well-defined fat-containing mass centered in the right parotid gland, arising from the deep lobe with lateral extension toward the deep portion of the superficial lobe, and showing medial exophytic extension into the right parapharyngeal space. (**b**) Axial contrast-enhanced CT shows heterogeneous enhancement with a discrete enhancing solid component along the medial aspect (arrows).

**Figure 2:**
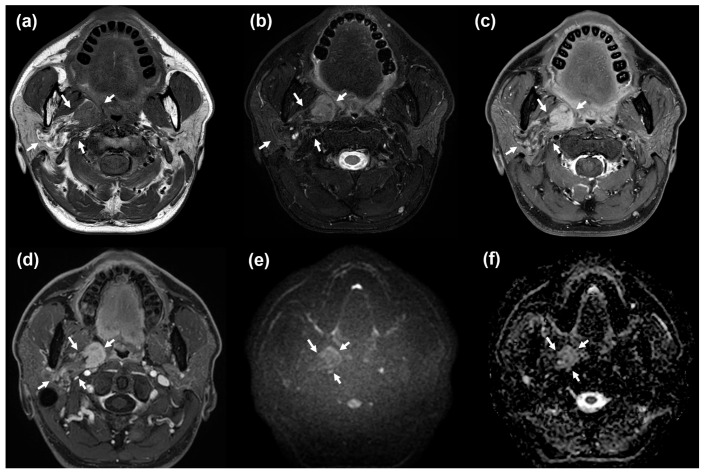
**MRI findings.** (**a**) Axial T1-weighted image shows a fat-containing mass centered in the right parotid gland, arising from the deep lobe with medial exophytic extension into the right parapharyngeal space, and a discrete solid portion along the medial aspect (arrows). The lobulated mass is clearly demarcated from the adjacent parapharyngeal fat plane. (**b**) Axial fat-suppressed T2-weighted image shows tiny hyperintense cystic foci within the lesion. (**c**,**d**) Axial fat-suppressed contrast-enhanced T1-weighted images at different levels show heterogeneous enhancement of the solid portion (arrows). (**e**,**f**) Axial diffusion-weighted image (DWI) and the corresponding apparent diffusion coefficient (ADC) map demonstrate mild diffusion restriction in the solid portion (arrows).

A 46-year-old man presented with a slowly growing palpable mass in the right parotid region that had been noticed approximately 2 years earlier. Physical examination revealed a non-tender, soft-tissue mass measuring approximately 3–4 cm in the mastoid tip area without overlying skin changes. The patient had no remarkable past medical history. Routine laboratory test results were normal. Contrast-enhanced neck CT demonstrated a well-circumscribed, lobulated, fat-containing mass with heterogeneous enhancement in the right parotid gland. An enhancing solid component was observed in the medial portion of the tumor (Figure 1). On MRI, the lesion demonstrated mild diffusion restriction within the medial enhancing solid component and contained small cystic foci (Figure 2). The lesion was centered in the parotid deep lobe and showed a bidirectional growth pattern: the fatty component extended laterally toward the deep portion of the superficial lobe, whereas the medial solid component bulged exophytically into the parapharyngeal space. These portions were contiguous components of a single biphasic mass rather than two separate lesions. No aggressive features, such as surrounding infiltration or pathologic lymphadenopathy, were observed. Given the fat-containing nature of the lesion and the presence of a discrete enhancing non-fat component, the leading considerations were pleomorphic adenoma with extensive fat metaplasia, oncocytic lipoadenoma, and an atypical lipomatous tumor/well-differentiated liposarcoma-spectrum lesion (ALT/WDL).

**Figure 3:**
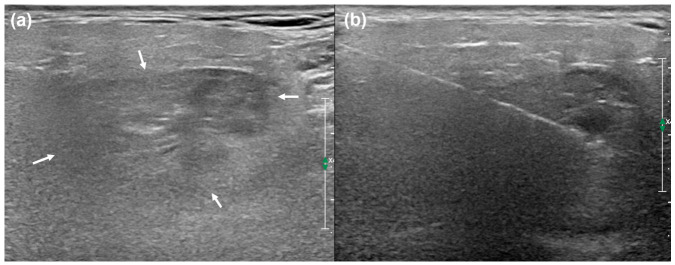
**US findings.** (**a**) Ultrasound shows a heterogeneously hypoechoic mass in the right parotid gland (arrows), with limited evaluation of the deep portion due to limited acoustic penetration. (**b**) An ultrasound-guided core-needle biopsy was performed targeting the fat component of the mass in the superficial lobe. The biopsy needle is visualized as a linear echogenic line in the image.

To further evaluate the lesion and obtain tissue samples, US was performed using a 5 to 12 MHz linear-array transducer. US images revealed a heterogeneously hypoechoic mass in the right parotid gland; however, limited acoustic penetration prevented adequate evaluation of the deep portion of the tumor. During the same session, an uncomplicated US-guided core-needle biopsy (CNB) was performed under local anesthesia with 1% lidocaine. The procedure was uneventful. Histopathological examination of the biopsy specimen suggested a lipomatous tumor; the vascular components were minimal, and no smooth muscle components were identified. Although overt lipoblasts were not clearly identified, an atypical lipomatous tumor could not be completely excluded based solely on the biopsy specimen (Figure 3).

**Figure 4:**
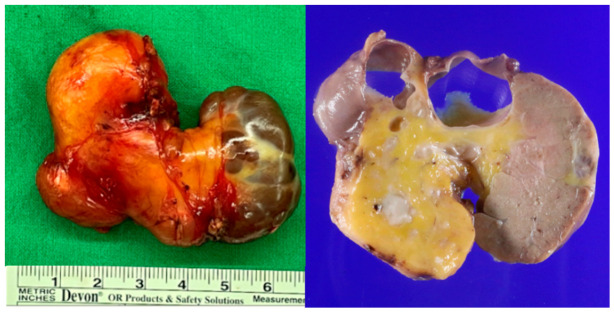
**Gross specimen.** A well-circumscribed, kidney-shaped, biphasic mass exhibits two cystic foci at the superior aspect and a fatty component on one side.

**Figure 5:**
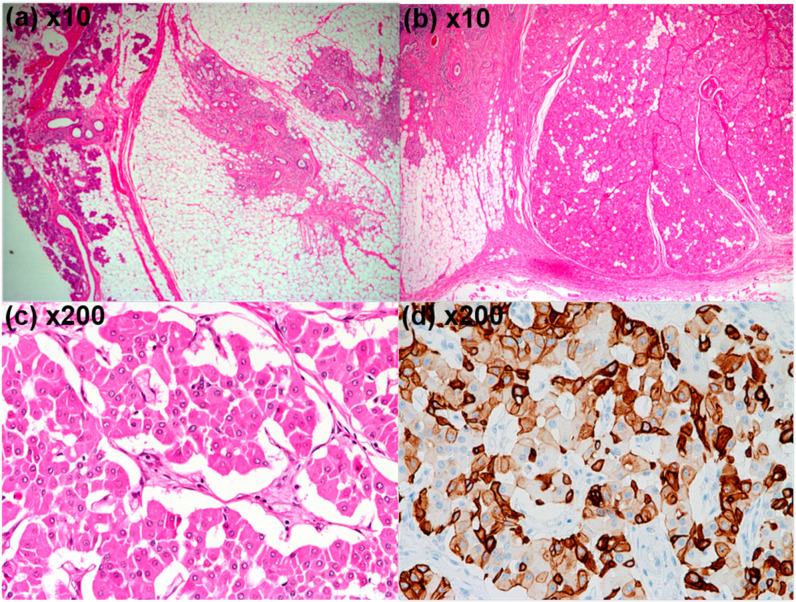
**Histopathologic findings.** (**a**–**c**) Hematoxylin and eosin (H&E) stained sections are shown. (**a**,**b**) Low-power views (×10) reveal the biphasic composition: adjacent salivary gland parenchyma with fatty replacement and periductal lymphocytic infiltration (**a**) is seen alongside eosinophilic trabeculae and sheets of oncocytic tumor nests intermingled with mature adipose tissue (**b**). (**c**) At high-power view (×200), large polygonal oncocytic cells are arranged in nested and trabecular patterns within the tumor. (**d**) Immunohistochemical staining (×200) demonstrates diffuse cytokeratin 7 positivity in the epithelial component.

Subsequent surgical excision was performed, and gross examination revealed a well-circumscribed, kidney-shaped, fat-containing tumor with two cystic foci, measuring 6.0 cm × 4.0 cm × 2.0 cm (Figure 4). Histopathological examination demonstrated eosinophilic trabeculae and sheets of tumor cell nests with oncocytic cells and mature adipose tissue, and the adjacent salivary gland parenchyma showed fatty replacement with periductal lymphocytic infiltration (hematoxylin and eosin [H&E], ×10) (Figure 5a,b). At higher magnification, large polygonal cells were arranged in nested and trabecular patterns within the tumor (H&E, ×200) (Figure 5c). Immunohistochemistry demonstrated cytokeratin 7 positivity (Figure 5d). Collectively, these findings supported the diagnosis of oncocytic lipoadenoma. The postoperative recovery was uneventful, and no recurrence was noted after 7 years of follow-up.

## 3. Discussion

This case demonstrates a practical multimodal imaging pattern for a fat-containing parotid mass—macroscopic fat with a discrete enhancing non-fat component—interpreted along with histopathological findings. To the best of our knowledge, only 27 cases of parotid oncocytic lipoadenoma have been reported across 16 publications (Table 1). Because this manuscript is a case report rather than a formal systematic review, the literature summary in Table 1 was constructed using targeted searches of PubMed and Google Scholar for previously reported cases, supplemented by a hand search of the reference lists of relevant articles. The search included the terms “oncocytic lipoadenoma” and “oncocytic sialolipoma.” Articles were included if they reported original parotid oncocytic lipoadenoma cases with extractable case-level clinical or pathologic information. Duplicate or overlapping cases, non-parotid lesions, and articles without sufficient case-level data were excluded from Table 1. For consistency with current salivary gland tumor nomenclature, this case report uses “oncocytic lipoadenoma” as the primary term, whereas “oncocytic sialolipoma” is mentioned only when referring to terminology used in earlier reports [1]. Table 1 was intended not as a chronological catalog, but as a concise contextual summary showing that most previous reports emphasized histopathologic or cytologic confirmation, whereas practical multimodal radiologic correlation across CT, MRI, and US has remained limited and inconsistent. Therefore, we present a component-oriented imaging assessment and highlight the specific contributions of this case.

Most previous reports on parotid oncocytic lipoadenomas have emphasized the rarity of the disease in cases confirmed primarily by postoperative histopathology, with limited preoperative imaging documentation. In contrast, our case illustrates that a multimodal imaging approach using CT, MRI, and US enables detailed characterization of lesion components and facilitates precise assessment of tumor location and extent within the parotid gland. This information was also clinically relevant for preoperative planning, because cross-sectional imaging clarified the deep-lobe origin, medial parapharyngeal extension, and preservation of adjacent fat planes, while the absence of overt aggressive features supported a lower suspicion for malignancy despite persistent diagnostic uncertainty on biopsy. Our experience suggests the usefulness of component-based assessment for evaluating fat-containing parotid masses in routine clinical practice. The initial step is to confirm true macroscopic fat and determine whether the finding is mass-forming rather than reflective of diffuse glandular fatty changes. Subsequent evaluations should focus on the non-fat component, particularly the discrete enhancing component, the overall architecture, and the absence of overt aggressive features, which may help prioritize benign biphasic entities over malignant lipomatous tumors. In the present case, macroscopic fat with a focally enhancing non-fat component supported a biphasic composition and favored a benign lesion on imaging. The lack of infiltrative margins, adjacent tissue invasion, or suspicious lymphadenopathy made a liposarcoma-spectrum lesion less likely, although it could not be fully excluded preoperatively. Accordingly, the leading considerations included pleomorphic adenoma with extensive fat metaplasia, oncocytic lipoadenoma, and a liposarcoma-spectrum tumor. More specifically, the differential diagnosis of a fat-containing parotid mass with a discrete enhancing non-fat component includes ordinary lipoma, sialolipoma, pleomorphic adenoma or myoepithelioma with extensive lipometaplasia, and rare atypical lipomatous tumor/well-differentiated liposarcoma-spectrum lesions [2]. A pure lipoma usually appears as a nearly homogeneous fatty lesion without a discrete enhancing epithelial component, whereas sialolipoma contains entrapped non-neoplastic salivary gland elements rather than a dominant oncocytic epithelial proliferation [2,14,15]. Pleomorphic adenoma with extensive lipometaplasia may also contain macroscopic fat, but it remains part of the broader morphologic spectrum of pleomorphic adenoma rather than a circumscribed oncocytic–adipocytic biphasic lesion [2]. In addition, when only the oncocytic component is sampled on fine-needle aspiration, oncocytoma or Warthin tumor may be suggested cytologically, particularly if the adipocytic component is scant or missed [9,13]. In our case, the combination of macroscopic fat, a discrete enhancing non-fat component, and the absence of infiltrative margins or suspicious lymphadenopathy favored a benign biphasic salivary lesion, with oncocytic lipoadenoma and pleomorphic adenoma with extensive fat metaplasia considered as the primary differential diagnoses. Although ALT/WDL cannot be excluded by a well-circumscribed margin alone, the absence of typical features such as thick septa (>2 mm), multiple or irregular nodular non-adipose components, and diffuse enhancement in our case made ALT/WDL less likely, favoring a benign biphasic salivary lesion [19,20].

This case also highlights the limitations of tissue sampling in patients with biphasic fat-containing parotid tumors. Because the fat component in oncocytic lipoadenomas can be unevenly distributed and variable in extent, core biopsy or fine-needle aspiration may be non-representative, potentially resulting in inconclusive pathological interpretation [15]. In our case, the fat component was dominant and predominantly located in the lateral aspect of the tumor, whereas the discrete enhancing solid component was situated more medially in the parotid deep lobe, extending into the parapharyngeal space. Limited acoustic penetration prevented confident visualization and targeting of this medial solid component; consequently, only the superficial fat-predominant portion was sampled, increasing diagnostic uncertainty and preventing complete exclusion of an atypical lipomatous tumor [21,22]. At the same time, US still played an important complementary role in the diagnostic process by serving as an accessible first-line modality, depicting the superficial portion of the lesion, and enabling real-time image-guided biopsy. However, this case also underscores that the diagnostic performance of US may be intrinsically limited in deep-lobe parotid tumors with medial parapharyngeal extension, particularly when the diagnostically important solid component cannot be confidently visualized or targeted. In such settings, comprehensive cross-sectional imaging is useful for delineating lesion architecture and stratifying concerns regarding malignancy. Therefore, a well-structured MRI protocol is recommended, including T1- and T2-weighted sequences with fat suppression (preferably Dixon techniques) and post-contrast fat-suppressed T1-weighted imaging to confirm macroscopic fat while depicting the enhancing non-fat component [23]. MRI is generally more useful than CT for preoperative characterization of parotid tumors because of its superior soft-tissue contrast [24,25]. In the present case, MRI more clearly depicted the biphasic architecture by confirming macroscopic fat, delineating the discrete enhancing non-fat component, and demonstrating tiny internal cystic foci within the solid portion, thereby allowing more refined characterization of lesion composition and a narrower differential diagnosis for a fat-containing parotid mass [24,25]. Diffusion-weighted imaging with apparent diffusion coefficient (ADC) mapping may further aid in characterizing the solid non-fat portion, particularly when the enhancing component is small or heterogeneous [26], and dynamic contrast-enhanced MRI may provide additional diagnostic value when available [27]. Recent multiparametric MRI studies further suggest that integrating conventional morphologic assessment with DWI/ADC and, when available, dynamic contrast-enhanced imaging and arterial spin labeling may improve preoperative characterization of parotid tumors [28]. By contrast, CT remains complementary, particularly for detecting calcification, evaluating osseous change or invasion, and in patients who cannot undergo MRI [24,27,29]. Quantitative dual-energy CT parameters may provide an additional complementary avenue for lesion characterization in selected patients [30]. Emerging deep learning-based ultrasound software and radiomics-based models are also promising adjuncts for lesion characterization, although current evidence remains predominantly retrospective and methodologically heterogeneous; thus, these software-based approaches are best regarded as complementary decision-support tools rather than routine standalone standards at present [31,32,33]. Overall, future improvement in preoperative characterization may depend on standardized multiparametric MRI, selective use of quantitative CT techniques, and software-based decision support integrated with careful radiologic-pathologic correlation and targeted sampling strategies.

From a histopathologic perspective, the diagnosis of oncocytic lipoadenoma is sup-ported not only by its characteristic biphasic architecture but also by its epithelial immunophenotype. Reported cases have shown that the oncocytic component typically ex-presses epithelial markers such as CK7, whereas myoepithelial markers, including calponin, are usually negative, supporting the absence of a true myoepithelial component [4,14,34,35]. In addition, p63 expression, when present, is generally limited to a small peripheral or basal-like cell population rather than being diffusely expressed in the oncocytic cells, which has been interpreted as partial basal-cell differentiation rather than true myoepithelial differentiation [4,14,34,35]. In our case, the diagnosis was supported by the characteristic biphasic morphology together with diffuse CK7 positivity in the epithelial component.

From a clinical standpoint, considering oncocytic lipoadenoma in the differential diagnosis may not eliminate the need for surgical excision of a growing parotid mass, but it can refine the preoperative assessment of malignancy risk and guide management planning. When imaging demonstrates macroscopic fat with a discrete enhancing non-fat component and no overt aggressive features, a benign biphasic process is a reasonable working diagnosis. This interpretation may help avoid overcalling malignancy and support appropriately tailored surgical planning and follow-up.

## 4. Conclusions

We described an exceptionally rare case of parotid oncocytic lipoadenoma presenting as a fat-containing parotid mass, supported by multimodal imaging and histopathological findings. In practice, awareness of the imaging pattern of macroscopic fat with a discrete enhancing non-fat component may help narrow the differential diagnosis and support more appropriate preoperative assessment of fat-containing parotid masses.

## Figures and Tables

**Table 1:** Reported cases of parotid gland oncocytic lipoadenoma.

Reported Cases (First Author, Year, [Ref])	Age (Years)	Sex	Laterality	Size (cm)	CT	MRI	US	Brief Reported Imaging Findings
Kato M, 2000 [3]	57	F	R	4.5	−	−	−	Not available
Klieb HB, 2006 [4]	46	F	L	3	−	−	−	Not available
Aouad R, 2008 [5]	38	M	L	3.8	+	+	−	CT: fat-containing mass with homogeneous enhancement of the non-fat component * Note—No representative image of MRI, but briefly described as an enhancing heterogeneous mass
Chahwala Q, 2009 [6]	50	F	L	14	−	−	−	Not available
Ilie M, 2010 [7]	64	M	L	5	+	−	−	No representative image of CT, but briefly described as a low-density heterogeneous mass
McNeil ML, 2010 [8]	73	M	L	4.2	−	−	−	Not available
Tokyol C, 2010 [9]	56	M	L	7	+	−	−	No representative image of CT, but briefly described as a well-circumscribed solid parotid mass
Devadoss CW, 2012 [10]	50	F	L	13.5	−	−	−	Not available
Mitsimponas KT, 2012 [11]	55	F	L	2.7	−	+	−	MRI: well-defined lesion with heterogeneous T1 signal intensity, without definite macroscopic fat * Note—Only axial T1WI and axial fat-saturated T2WI were available
Agaimy A, 2013 [12]—case 1	63	M	L	4.5	−	−	−	Not available
Agaimy A, 2013 [12]—case 2	29	M	L	4.5	−	−	−	Not available
Agaimy A, 2013 [12]—case 3	54	F	NS	2.9	−	−	−	Not available
Agaimy A, 2013 [12]—case 4	7	F	L	NS	−	−	−	Not available
Agaimy A, 2013 [12]—case 5	89	F	NS	4.2	−	−	−	Not available
Agaimy A, 2013 [12]—case 6	55	M	NS	2.7	−	−	−	Not available
Ashraf MJ, 2015 [13]	56	F	R	3	−	−	−	Not available
Chi CL, 2015 [14]	71	M	R	4.2	+	−	−	CT: heterogeneous attenuation with a fatty component * Note—The available images were limited in resolution and technical detail
Lau SK, 2015 [15]—case 1	61	M	L	2	−	−	−	Not available
Lau SK, 2015 [15]—case 2	83	M	R	2.5	−	−	−	Not available
Lau SK, 2015 [15]—case 3	67	M	R	4	−	−	−	Not available
Lau SK, 2015 [15]—case 4	40	F	R	4	−	−	−	Not available
Lau SK, 2015 [15]—case 5	56	M	L	3.5	−	−	−	Not available
Lau SK, 2015 [15]—case 6	65	M	L	1.9	−	−	−	Not available
Lau SK, 2015 [15]—case 7	65	M	R	3.5	−	−	−	Not available
Shakya D, 2020 [16]	46	M	R	15	+	−	−	CT: fat-containing solid mass with enhancement, without calcification or cystic component * Note—The available images were limited in resolution and technical detail
Sureja VP, 2023 [17]	59	M	R	5.5	−	−	−	Not available
Alotaibi JK, 2024 [18]	69	M	R	11.5	−	+	−	MRI: discrete soft-tissue mass with heterogeneous T2 signal intensity * Note—Only axial and sagittal fat-saturated T2-weighted images were available
Present case	46	M	R	6.0	+	+	+	CT: lobulated fat-containing mass with medial enhancing solid component; MRI: mild diffusion restriction and small cystic foci; US: heterogeneously hypoechoic mass with limited deep evaluation

* Note—“+” indicates that the modality was reported/performed in the source article; “−” indicates that the modality was not reported or that representative imaging was not available in the source article. F, female; L, left; M, male; NS, not specified; R, right.

## Data Availability

The original contributions presented in this study are included in the article material. Further inquiries can be directed to the corresponding author..

## Abbreviations

The following abbreviations are used in this manuscript:

ADC	Apparent diffusion coefficient
ALT/WDL	Atypical lipomatous tumor/well-differentiated liposarcoma-spectrum lesion
CK7	Cytokeratin 7
CT	Computed tomography
H&E	Hematoxylin and eosin
MRI	Magnetic resonance imaging
US	Ultrasonography
